# Anion-Controlled Inorganic Materials as Catalysts for Small-Molecule Conversion Reactions

**DOI:** 10.1021/acsami.5c00825

**Published:** 2025-04-17

**Authors:** Megumi Okazaki, Yuta Tsuji, Daisuke Tanaka, Hiroshi Kageyama, Kazuhiko Maeda

**Affiliations:** † School of Science, Institute of Science Tokyo, 2-12-1-NE-2 Ookayama, Meguro-ku, Tokyo 152-8550, Japan; ‡ Faculty of Engineering Sciences, Kyushu University, Kasuga, Fukuoka 816-8580, Japan; § Department of Chemistry, School of Science, Kwansei Gakuin University, Gakuen-Uegahara, Sanda, Hyogo 669-1337, Japan; ∥ Department of Energy and Hydrocarbon Chemistry, Graduate School of Engineering, Kyoto University, Nishikyo-ku, Kyoto 615-8510, Japan; ⊥ Research Center for Autonomous Systems Materialogy (ASMat), Institute of Science Tokyo, 4259 Nagatsuta-cho, Midori-ku, Yokohama, Kanagawa 226-8501, Japan

**Keywords:** artificial photosynthesis, coordination polymers, heterogeneous catalysis, metal−organic frameworks, mixed-anion compounds, photocatalysis

## Abstract

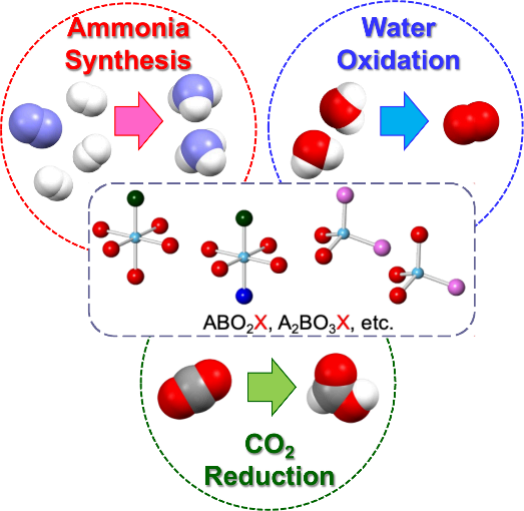

Various catalytic reactions such as water oxidation to O_2_ and CO_2_ reduction have been achieved using the surfaces of inorganic materials as reaction sites, as exemplified by oxygen-defect-induced reactions in metal oxides. In recent years, anion-controlled post-transition-metal oxide materials have attracted attention. These materials have been shown to exhibit unique reactivity that cannot be achieved with oxides because of the effect of paired anion species coexisting with oxide anions. They have also been shown to enable highly difficult reactions because of the surface and reaction properties resulting from the molecular nature of the anion species. This Perspective reports on recent developments in small-molecule conversion reactions catalyzed by anion-controlled inorganic materials.

## 1 Introduction

The surface of metal oxides is known to serve as a platform for the catalysis for various small-molecule transformations, such as water splitting and CO_2_ conversion. The catalytic activities depend strongly on the local structure of the metal–oxygen coordination polyhedral units and the electronic state of the central metal cations. For instance, the number of *d*-electrons in the metal cation, which determines the bonding state with reaction intermediates, is known to strongly influence the catalytic activity toward the electrochemical oxidation of water to molecular O_2_.^1^ Recently, high-entropy metal oxides, which incorporate more than five cationic elements, have also been reported to exhibit high catalytic activity for the electrochemical O_2_ evolution reaction (OER).^2,3^ However, the types of achievable coordination polyhedral units are limited to those of the metals and the crystal structure; cation-dependent control of the electronic state of the active metal center is also limited.

**Scheme 1:**
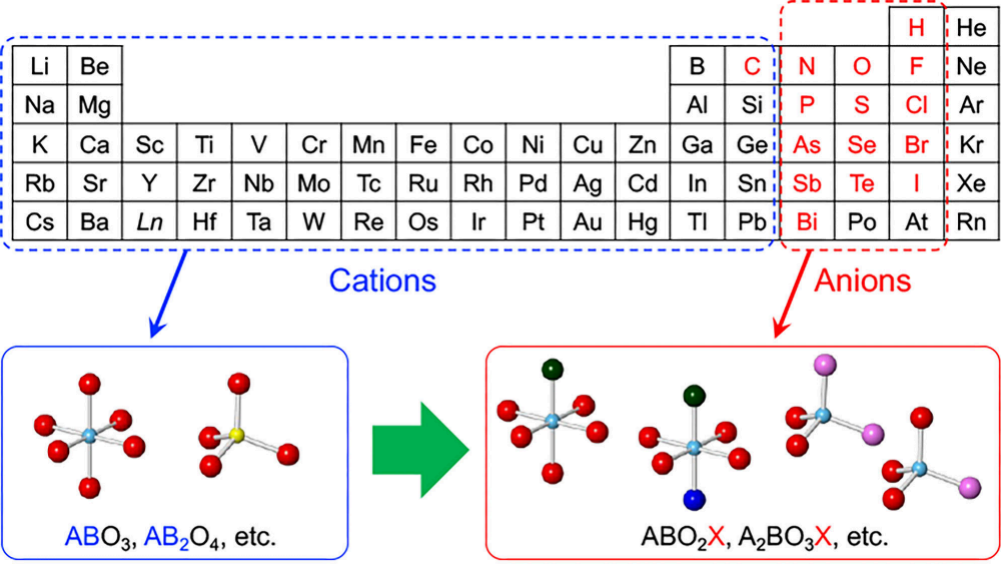
From Cation- to Anion-Controlled Inorganic Materials Based on a Mixed-Anion Strategy

In this context, “mixed-anion compounds”, in which more than one anionic species coexist within the same single-phase compound, have attracted attention.^4−6^ Taking mixed-anion compounds based on oxides (e.g., oxynitrides and oxyhalides) as examples, anions with electronegativities that differ from that of oxygen form various metal–anion coordination polyhedral units along with metal–oxygen bonds (see Scheme 1), thereby substantially modulating the electronic state of the metal. Incorporating the essence of anion chemistry into established cation-based inorganic materials enables the development of functional materials with outstanding properties and functions. In practice, oxynitrides and oxysulfides with anions less electronegative than oxygen can form valence bands at the high-energy sides because of the low electronegativity of these anions. As a result, they are useful as visible-light-responsive photocatalysts, with active research being conducted since approximately 2000.^7−9^ In addition, over the past decade, anion species and/or defects derived from them have been reported to directly participate in surface chemical reactions. In some cases, the unique electronic states of metal cations created by the coexistence of multiple anions provide intriguing catalytic activity.

**Scheme 2:**
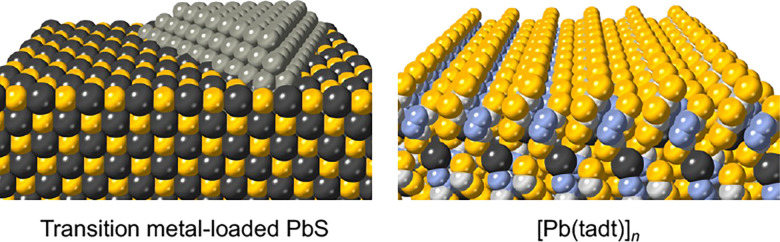
Comparison of the Surface Structures of Inorganic Materials The left diagram shows a transition-metal-loaded PbS surface, whereas the right diagram shows an unloaded, clean surface of a Pb–S-based coordination polymer [Pb(tadt)]*_n_* (tadt^2–^ = 1,3,4-thiadiazole-2,5-dithiol). Black, Pb; yellow, S; light gray, C; blue, N.

Another intriguing research target is a group of compounds composed of molecular anions connected by covalent bonds.^10^ Unlike compounds consisting of monatomic anions that provide an essentially flat surface, inorganic compounds formed by large molecular anions can exhibit unique surface structures with neighboring cations, potentially serving as excellent reaction sites for small-molecule conversion reactions (Scheme 2). Such a landscape may be common in the context of homogeneous catalysis involving primarily single molecules; however, from the perspective of heterogeneous catalysis on solid surfaces, it appears to offer a new possibility.

This Perspective provides an overview of unique surface chemical reactions realized by anion-controlled inorganic materials (e.g., mixed-anion compounds) in recent years, with a focus on small-molecule conversions, including ammonia synthesis, water splitting, and CO_2_ reduction. As mentioned above, certain mixed-anion compounds have been intensively studied as heterogeneous photocatalysts for visible-light water splitting and CO_2_ reduction, with review papers that focus on specific topics already available.^7−9^ In such visible-light-responsive mixed-anion photocatalysts, the anion species play a key role in enabling visible-light absorption but do not directly participate in promoting surface reactions. Therefore, this Perspective does not discuss conventional research on visible-light-responsive mixed-anion photocatalysts but instead focuses on cases where anion species significantly contribute to chemical reactions.

## 2 Mixed-Anion Materials for Catalysis

### 2.1 Oxyhydrides for Ammonia Synthesis

Ammonia synthesis from H_2_ and N_2_ is a crucial process supporting billions of people globally. As described below, the reaction is exothermic, favoring low temperatures and high pressures, with catalysts being essential for its efficient operation:
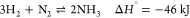


The Haber–Bosch process, established over a century ago, uses a doubly promoted Fe-based catalyst (Fe_3_O_4_–Al_2_O_3_–K_2_O). Since the 1970s, research has led to second-generation catalysts, such as an Ru-based catalyst on activated carbon, which exhibits enhanced activity. However, current processes still require high temperatures (573–773 K) and pressures (150–300 atm), consuming substantial energy. Thus, an urgent need exists for more efficient catalysts that function under milder conditions.

**Figure 1:**
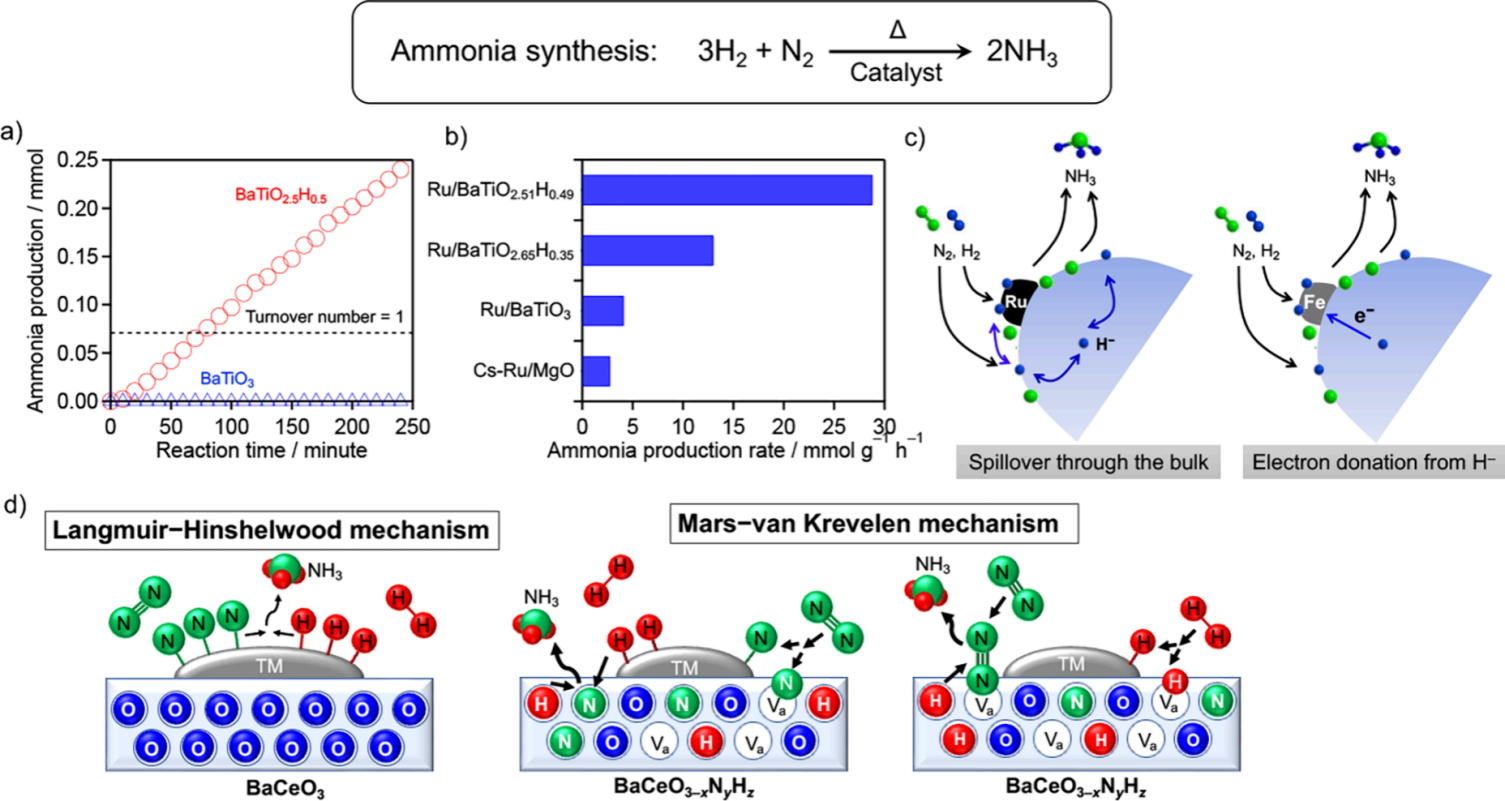
Ammonia synthesis using mixed-anion catalysts: a) Time course of ammonia generation reactions on BaTiO_3_ and BaTiO_2.5_H_0.5_ (50 atm, 673 K). Reproduced from ref (12) with permission. Copyright 2017 American Chemical Society. b) Ammonia generation reactions on various supported Ru catalysts (Ru 1 wt % (nominal amount), 50 atm, 673 K). Reproduced from ref (14) with permission. Copyright 2018 Wiley VCH. c) Possible metal-dependent mechanisms for the high activity of metal-loaded BaTiO_3–*x*_H_*x*_ catalysts; a Ru or Fe particle (black or gray) is shown on a BaTiO_3–*x*_H_*x*_ support particle (large blue sphere). (left) Colossal spillover of H^–^ ions through a support particle (“bulk”), hindering H_2_ poisoning on Ru. (right) Partial electron donation from H^–^ to the Fe particle, assisting in N_2_ activation. Reproduced from ref.^14^ with permission. Copyright 2018 Wiley VCH. d) Possible reaction mechanisms for ammonia synthesis: the Langmuir–Hinshelwood mechanism, in which N_2_ and H_2_ react on the transition-metal (TM) catalyst supported on BaCeO_3_, and an anion-vacancy (Va)-mediated Mars–van Krevelen (MvK) mechanism over TM/BaCeO_3–*x*_N_*y*_H_*z*_, in which lattice N^3–^ and H^–^ ions participate in the ammonia synthesis reaction. Reproduced from ref (15) with permission. Copyright 2019 American Chemical Society.

Recently, the Ti-based oxyhydride perovskite BaTiO_2.5_H_0.5_ typically, synthesized by a topochemical reaction of BaTiO_3_ with CaH_2_ in an evaluated glass tube at ∼853 K followed by washing with NH_4_Cl–methanol solution,^11^ was found to exhibit catalytic activity for ammonia synthesis even without the aid of a transition-metal catalyst.^12^ As shown in Figure 1a, whereas the corresponding oxide BaTiO_3_ exhibited no activity under the same reaction conditions, BaTiO_2.5_H_0.5_ operated stably over a long period. The amount of ammonia produced in the catalytic activity test was substantially greater than the theoretical amount based on the assumption that all hydrogen in BaTiO_3–*x*_H*_*x*_* is consumed, clearly indicating that the ammonia synthesis reaction proceeded catalytically due to the effect of H^–^ ions in the perovskite crystal lattice. The ability of the oxyhydride to activate N_2_ molecules is evident from the formation of the corresponding oxynitride when BaTiO_2.5_H_0.5_ was treated with N_2_ gas without H_2_ gas. This high reactivity of Ti–H bonds is believed to promote the dissociation of stable N_2_ molecules, leading to catalytic activity for ammonia synthesis. Subsequently, a general understanding has been established that even compounds containing several early transition metals, such as V and Ti (which are usually considered unsuitable as ammonia synthesis catalysts because of their strong binding affinity with N_2_), can function as excellent catalysts through the introduction of hydrides.^13^

The ammonia synthesis activity was found to be further enhanced by introducing transition metals such as Ru, Fe, or Co as active sites onto a BaTiO_3–*x*_H*_*x*_* (0.3 ≤ *x* ≤ 0.6) support.^14^Figure 1b shows the results of a comparison of the catalytic activity of a catalyst with Ru 1 wt % loading at 50 atm and 673 K. As evident from these results, Ru/BaTiO_2.5_H_0.5_ demonstrated substantially higher activity than conventional catalysts such as Cs–Ru/MgO and Ru/BaTiO_3_ and operated stably for 48 h without the addition of a reaction promoter. For Ru/BaTiO_2.5_H_0.5_ and Ru/BaTiO_3_, the temperature and pressure dependence of the ammonia production rate have been investigated, respectively. In the temperature/pressure range examined (573–673 K at 5 MPa and 0.1–5 MPa at 673 K), Ru/BaTiO_2.5_H_0.5_ showed higher activity than Ru/BaTiO_3_. Although BaTiO_2.5_H_0.5_ without a loaded metal also acted as an active catalyst, the loading of metal was essential for achieving high activity. The catalytic activity was also found to depend on the hydride content in BaTiO_3–*x*_H*_*x*_*, with a higher hydride content generally leading to greater activity.

The key characteristic of the Ru/BaTiO_3–*x*_H*_*x*_* catalyst is its efficient ammonia production under pressurized conditions. From a thermodynamic perspective, increasing the pressure of the feed gas should enhance the rate of ammonia production; however, for many Ru catalysts, including Ru/BaTiO_3_, the ammonia production rate does not increase with increasing reaction pressure because the Ru surface becomes poisoned by hydrogen, which inhibits N_2_ dissociation. By contrast, Ru/BaTiO_2.5_H_0.5_ exhibits improved activity in the pressure range 1–50 atm, showing higher activity than Ru/BaTiO_3_ across the entire pressure range. This behavior strongly suggests that hydrogen poisoning does not occur on Ru/BaTiO_2.5_H_0.5_. Here, the reaction rate (*r*) for ammonia production is given by the equation
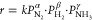
where *k* is the reaction rate constant; *P*_N_2__, *P*_H_2__, and *P*_NH_3__ represent the partial pressures of N_2_, H_2_, and ammonia, respectively; and α, β, and γ are the corresponding reaction orders. Detailed kinetic analysis experiments in which the partial pressures of N_2_, H_2_, and ammonia were varied confirmed that the reaction order β for H_2_ on the Ru/BaTiO_2.5_H_0.5_ catalyst is positive (0.2 ± 0.1). This result is in contrast to the negative reaction order commonly observed for many Ru catalysts, indicating that this catalyst can produce ammonia despite hydrogen poisoning (e.g., −1.5 ± 0.2 for Cs–Ru/MgO). As previously mentioned, H^–^ anions in BaTiO_3–*x*_H*_*x*_* have been shown to activate small molecules in the gas-phase atmosphere, enabling anion-exchange reactions to occur at moderate temperatures (<723 K). For example, in a D_2_ gas flow, BaTiO_3–*x*_D*_*x*_* can be obtained via H/D exchange, and in a N_2_ gas flow, BaTiO_3–*x*_N_2*x*/3_ is formed through H/N exchange.^16^ Such partial anion-exchange reactions also occur during ammonia synthesis from N_2_ and H_2_, resulting in a triple-anion oxide–hydride–nitride BaTi(O,H,N)_3−δ_. During this process, hydrogen adsorbed onto Ru is transferred into the perovskite lattice as H^–^ ion, which subsequently diffuses through the perovskite lattice. This colossal spillover is thought to prevent hydrogen poisoning of the Ru catalyst by enabling the H^–^ ions to migrate away from the metal surface.

Like BaTiO_2.5_H_0.5_, TiH_2_ is also known to exhibit high catalytic activity for ammonia synthesis.^12^ On the surface of TiH_2_, hydrogen vacancies are expected to form and regenerate repeatedly, enabling ammonia synthesis via a unique hydrogen-based Mars–van Krevelen (MvK) mechanism. The work function of TiH_2_ is as low as that of metallic Ti, facilitating efficient electron injection into N_2_ molecules, promoting the dissociation of N_2_ molecules on its surface.^17^ Consequently, the rate-determining step is thought to shift toward the formation process of N–H bonds. In addition, it has been noted that the formation of N–H bonds is promoted by a synergistic effect between nitrides penetrating beneath the surface of TiH_2_ and hydrides present on its surface.

Another effect of H^–^ anions in oxyhydride supports is electron donation to the supported metal species, as demonstrated by the relatively low activation energy and α for Fe/BaTiO_2.4_H_0.6_.^14^ In general, support-to-metal electron donation weakens the triple bond of an adsorbed N_2_ molecule and the cleavage of the triple bond is considered to be the rate-determining step for ammonia synthesis. Therefore, the low activation energy for Fe/BaTiO_2.4_H_0.6_ (54–73 kJ mol^–1^) indicates a strong electron-donation effect from the oxyhydride support. The reaction order of N_2_ for Fe/BaTiO_2.4_H_0.6_ (0.5 ± 0.1) was approximately one-half of those for conventional catalysts (e.g., 1.14 ± 0.08 for Cs–Ru/MgO). These results led to the conclusion that the electron donation from the oxyhydride support enables the adsorbed N_2_ molecules to dissociate more smoothly on the Fe surface, with the subsequent surface reaction of dissociated N and H atoms being the rate-limiting step. Thus, oxyhydride supports, which provide a hydrogen-based MvK mechanism with a lower frequency of hydrogen poisoning and reinforced electron-donating capabilities for the supported metal, have been shown to be critically important for enhancing the catalytic activity toward ammonia synthesis (Figure 1c). A similar electron donation effect has been reported for Ru-loaded Ba*Re*O_2_H (*Re* = Sc, Y) catalysts.^18^

As another mixed-anion catalyst for ammonia synthesis, the perovskite oxynitride-hydride BaCeO_3–*x*_N*_*y*_*H*_*z*_* was synthesized directly at 573–873 K by the reaction of CeO_2_ and Ba(NH_2_)_2_.^15^ BaCeO_3–*x*_N*_*y*_*H*_*z*_* was found to function as an efficient catalyst for ammonia synthesis and to work according to the MvK mechanism via N^3–^ and H^–^ ions in its crystal lattice, irrespective of supported metals (Figure 1d). The activation energy for ammonia synthesis is 46–62 kJ mol^–1^, which is approximately one-half of the activation energy of conventional catalysts, including BaCeO_3_, driven by the Langmuir–Hinshelwood mechanism (85–121 kJ mol^–1^).

### 2.2 Oxyfluorides for the Oxygen Evolution Reaction

The OER is the key reaction to achieve artificial photosynthesis processes such as ammonia formation, CO_2_ reduction, and H_2_ generation for green energy. O_2_ evolution from water involves four electrons, as described below; therefore, the reaction mechanism itself is quite complicated.
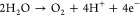


Noble-metal oxides such as IrO_2_ and RuO_2_ exhibit high activity as OER catalysts; however, the development of catalysts composed of earth-abundant elements is currently needed. In recent years, several oxyfluorides containing first-row transition metals have been reported to function as OER electrocatalysts. In addition, the electrocatalytic activity of some of these materials has been found to be enhanced by the combination of multiple transition metals. The incorporation of F into a structure composed of multiple early transition metals and oxygen is expected to lead to crystal structures that differ from those of conventional metal oxides, as well as to the ability to control the electronic states of metal–anion polyhedra.

**Figure 2:**
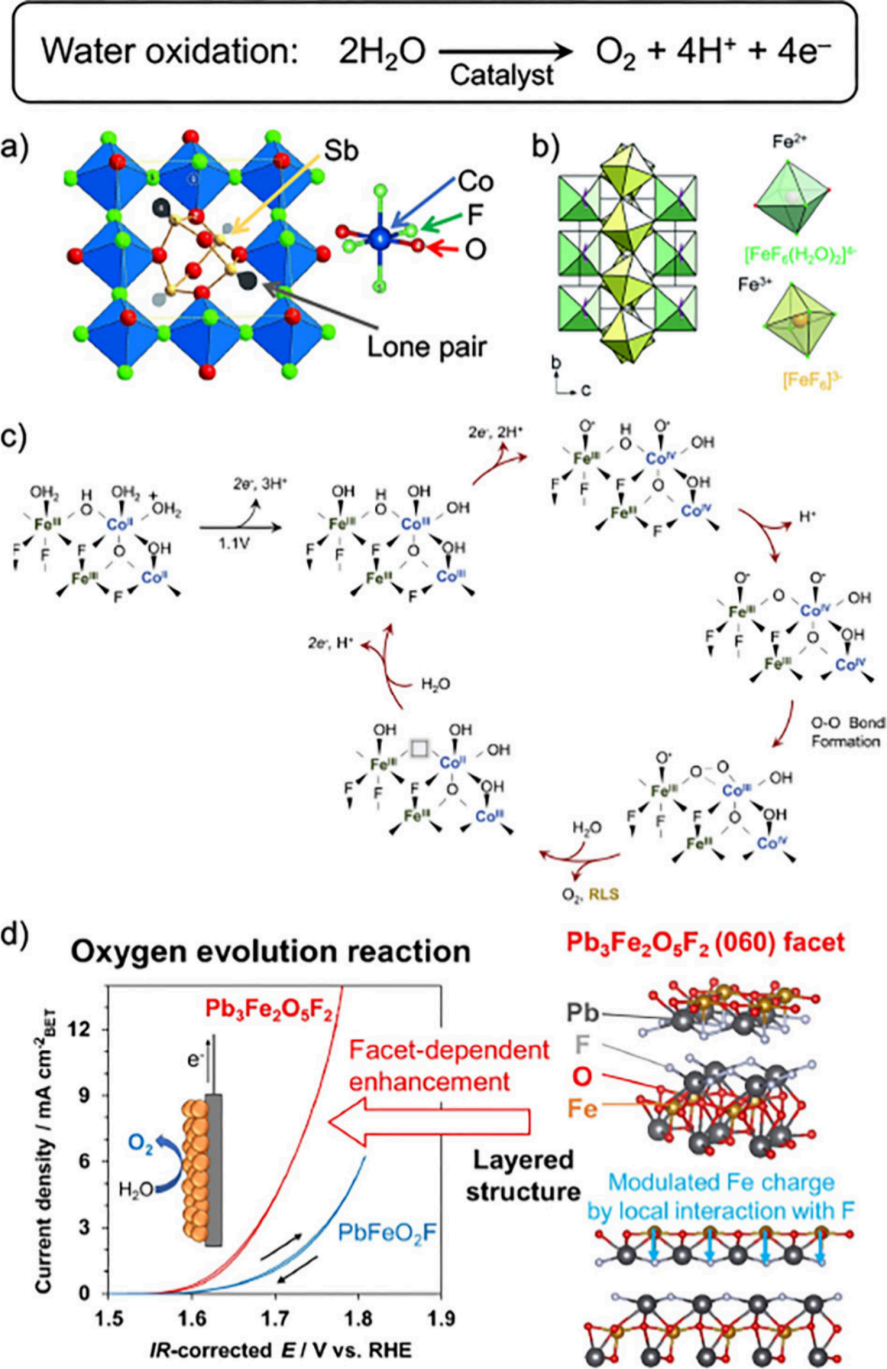
a) Structure of Co_3_Sb_4_O_6_F_6_, composed of Co (blue), O (red), Sb (yellow), a lone pair (gray), and F (green); it is made up of a slightly distorted [CoO_2_F_4_] octahedra that corner share via F atoms. Reproduced from ref (19) with permission. Copyright 2015 Wiley VCH. b) Structure of *M*Fe_2_F_8_(H_2_O)_2_ (*M* = Co, Ni). Reproduced from ref (21). c) Proposed mechanism of the OER over Co_0.5_Fe_0.5_O_0.5_F_1.5_. Surface charges are omitted for simplicity. Reproduced from ref (23) with permission. Copyright 2022 Cell Press. d) Cyclic voltammetry of electrocatalytic OER over the layered Pb_3_Fe_2_O_5_F_2_ and PbFeO_2_F, and a schematic of the role of F in the layered Pb_3_Fe_2_O_5_F_2_. Reproduced from ref (26).

Johnsson et al. reported that the oxyfluoride solid solution (Co*_*x*_*Ni_1–*x*_)_3_Sb_4_O_6_F_6_ exhibited OER catalytic activity, whereas Ni_3_Sb_4_O_6_F_6_ did not.^19^ The overpotential of (Co*_*x*_*Ni_1–*x*_)_3_Sb_4_O_6_F_6_ for the OER was lower than that of Co_3_Sb_4_O_6_F_6_. (Co*_*x*_*Ni_1–*x*_)_3_Sb_4_O_6_F_6_ has an octahedral [CoO_2_F_4_] coordination at the active metal center. This coordination is connected to the Sb via an oxo bridge, which is believed to contribute to its catalytic function (Figure 2a). Similar to the catalytic mechanism of photosystem II in nature, an F atom and its lone pair on the catalyst surface directs the reactant species to preferred sites although F itself does not participate in the reaction. The Sb–O bond, which is part of the oxo bridge, has a flexible coordination and is believed to contribute to the promotion of the OER.

Fe is another featured element in oxyfluoride-type OER electrocatalysts. Habazaki et al. reported that Fe-doped Ni(OH)_2_ deposited onto a Ni substrate by in situ anodization showed enhanced electrochemical activity when a F^–^-containing solution was used as the electrolyte.^20^ Kornienko et al. showed that amorphous mixed-metal oxyfluorides prepared from hydrated fluorides can exhibit stable electrochemical OER activity.^21,22^ They prepared the hydrated fluoride precursor via microwave-assisted solvothermal synthesis. Fe combined with Co in the form of CoFe_2_F_6.6_O_0.7_ prepared from CoFe_2_F_8_(H_2_O)_2_, along with Fe combined with Ni in the form of NiFe_2_F_4.4_O_1.8_ prepared from NiFe_2_F_8_(H_2_O)_2_ (Figure 2b), showed high electrochemical OER performance with a relatively low overpotential (270 mV at 10 mA cm^–2^) and good stability (>250 h) in alkaline media. However, amorphous manganese–iron oxyfluoride materials (MnFeF_4.6_O_0.2_ and MnFe_2_F_5.8_O_1.1_) obtained from crystalline–hydrated fluorides showed high stability in acidic media compared with as-reported Mn-based water oxidation catalysts. By changing the element paired with Fe, one could obtain a series of cost-effective and earth-abundant oxyfluoride materials as OER electrocatalysts that function under a wide range of pH conditions. The Co–Fe mixed oxyfluoride Co_0.5_Fe_0.5_O_0.5_F_1.5_, which was obtained by heating the corresponding hydrated fluoride precursor, was reported to exhibit very low OER overpotentials of 220 mV and 265 mV at current densities of 10 mA cm^–2^ and 100 mA cm^–2^, respectively.^23^ On the basis of the performance metrics of overpotential, Tafel slope, mass activity, and stability, the authors claimed that the Co_0.5_Fe_0.5_O_0.5_F_1.5_ catalyst was one of the best-performing Co-based OER electrocatalysts ever reported. The redox cycle in metal species was confirmed only at the Co site, whereas the Fe site typically did not show any redox behavior. The authors also confirmed through a comparison with a cobalt–iron oxide prepared by postheating the oxyfluoride that F plays a critical role in the improvement of electrocatalytic activity. From the viewpoint of both experimental and theoretical approaches, a Co–Fe bimolecular site was determined to be an active site for the catalytic reaction. The mechanism is similar to the ″lattice-oxygen-mediated″ (LOM) mechanism, in which the lattice oxide anions are involved in the OER, and this participation of lattice oxide anions is one of the factors contributing to its high activity (Figure 2c). At the same time, the high stability of the Co_0.5_Fe_0.5_O_0.5_F_1.5_ catalyst suggests that the OER mechanism does not fully follow the LOM. Therefore, F is considered to play an important role in the formation and maintenance of the structure of the catalyst even though it does not appear to be involved in the OER mechanism itself. In summary, the electrocatalytic OER performance of oxyfluorides is likely strongly affected by their surface structure.

More detailed roles of F in the oxyfluoride crystal in the electrochemical OER were revealed using two different oxyfluoride perovskites Pb_3_Fe_2_O_5_F_2_ and PbFeO_2_F, which were synthesized in vacuo at 873 K and under high pressure (6.0 GPa) at 1173 K, respectively, through a conventional solid-state reaction using the corresponding oxide and/or fluoride precursors.^24,25^ It was clarified that the layered perovskite Pb_3_Fe_2_O_5_F_2_ exhibits a lower overpotential and higher current density for the OER than the bulk-type cubic three-dimensional perovskite PbFeO_2_F.^26^ The electrochemical performance of Pb_3_Fe_2_O_5_F_2_ was also slightly improved by amorphization of the surface of the electrode during electrochemical measurements, and it was found to be stable for longer than 18 h. A small level of Pb^2+^ leaching from Pb_3_Fe_2_O_5_F_2_ was observed during the operation, but it did not adversely affect the OER activity. Density functional theory (DFT) calculations showed that the (060) facet in Pb_3_Fe_2_O_5_F_2_ diminishes the overpotential for water oxidation because of the moderately strong interaction between the active sites and the reaction intermediates (Figure 2d); that is, the relevant interaction was found to be enhanced by the strong electron-withdrawing ability of F^–^ ions.

In addition, recent studies have revealed that oxychloride compounds can also act as OER catalysts. Layered perovskites of Sr_4_Fe_3_O_8_Cl_2_ and Sr_2_FeO_2_Cl_2_ were found to exhibit OER (and oxygen reduction reaction (ORR)) activity in alkaline media.^27^ More recently, the Ruddlesden–Popper-type oxychloride Sr_2_Co_0.8_Fe_0.2_O_3_Cl was found to catalyze both the OER and the ORR.^28^ The fact that this material demonstrates higher activity than the metal oxide Ba_0.5_Sr_0.5_Co_0.8_Fe_0.2_O_3−δ_ (BSCF), which is known as one of the best-performing OER electrocatalysts, strongly supports the idea that oxychlorides also exhibit good OER catalytic performance. The free energy calculation results assuming a single site as the active site cannot explain the high activity of Sr_2_Co_0.8_Fe_0.2_O_3_Cl. However, when Co and Fe were considered as dual-active sites, the authors argued that passing through the Co-OOH and Fe-OOH intermediates makes the system more favorable for high OER activity from the perspective of free energy. Therefore, it can be concluded that the high activity was achieved by Co and Fe alternately functioning as active sites.

## 3 Nonporous Coordination Polymers for Efficient CO_2_ Reduction

Coordination polymers (CPs) and metal–organic frameworks (MOFs) are considered to have strong potential as catalysts and/or catalyst supports because of their effective uptake of reaction substrates, which is attributed to their porous structures and high specific surface areas.^29,30^ Along this line, the visible-light-driven reduction of CO_2_ using CPs/MOFs as photocatalysts has been reported.^31^ Although several examples of visible-light CO_2_ reduction photocatalysts based on CPs/MOFs have shown relatively good activity, many of these photocatalysts require postsynthesis modification to enhance visible-light absorption and introduce catalytic active sites, thereby fully realizing their photocatalytic potential.

By contrast, from a solid-state chemistry perspective, CPs/MOFs contain molecular anionic species with a rich elemental diversity. Unique reactivity arising from these molecular anions present on their surface is anticipated. Moreover, the crystalline surfaces of CPs/MOFs composed of molecular anions are expected to differ fundamentally from the inherently flat surfaces of conventional metal oxides or mixed-anion compounds, which consist of monatomic ions. From this viewpoint, the surfaces of CPs/MOFs can provide a good platform for the transformation of small molecules.

Given this background, a CP composed of Pb^2+^ and 1,3,4- thiadiazole-2,5-dithiol (H_2_tadt), [Pb(tadt)]*_n_* (named KGF-9), has been identified as a good photocatalyst for CO_2_ reduction.^32^ Although most CPs/MOFs are ionic insulating solids, KGF-9 forms a conduction band with Pb and tadt, resulting in an electronic structure similar to that of conventional semiconductors, which are photoconductive. In addition, molecular-reaction-active sites are formed on the KGF-9 crystal surface, resulting in properties intermediate between those of solids and molecules and manifesting unique catalytic characteristics.

**Figure 3:**
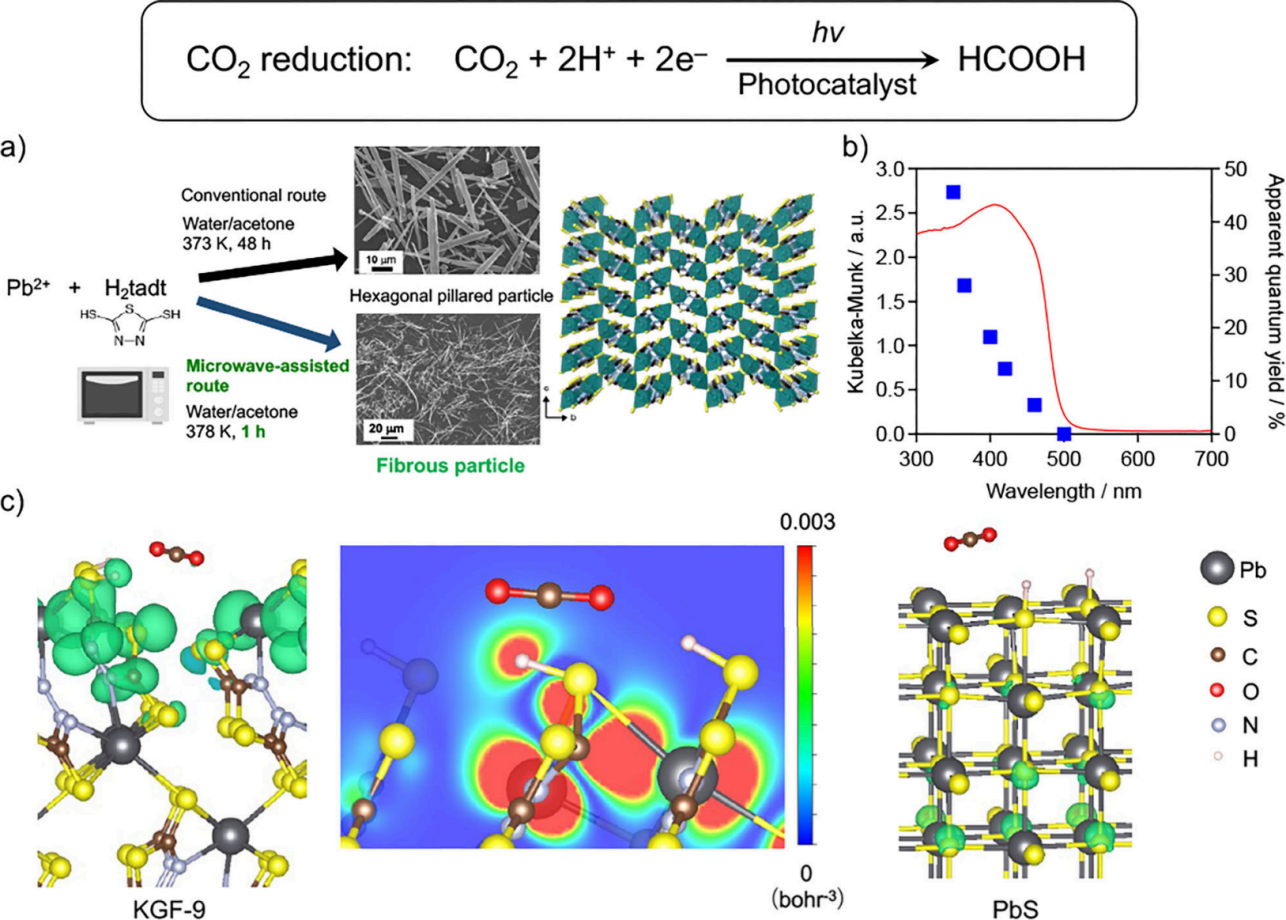
CO_2_ reduction using coordination polymer photocatalysts. a) Synthesis of [Pb(tadt)]*_n_* (KGF-9). b) UV–vis diffuse-reflectance spectra of the optimized KGF-9 synthesized by a microwave-assisted solvothermal method, plotted, along with the apparent quantum yields for formate production over KGF-9, as a function of wavelength. Reaction conditions: photocatalyst, 10 mg; DMSO, 15 mL; electron donor, 0.1 M BIH; light source, 300 W Xe lamp (λ = 350, 365, 400, 420, 460, 500 nm); light intensity, 0.4 mW cm^–2^. Reproduced from ref (33). c) Isosurface and two-dimensional charge density map just below the Fermi level on the KGF-9 surface (left) and the corresponding isosurface on the PbS surface (right). The CO_2_ molecule is trapped in a concave region of the KGF-9 surface, with the charge density distributed around it. Reproduced from ref (34) with permission. Copyright 2024 American Chemical Society.

Figure 3a shows the synthetic scheme and crystal structure of KGF-9. In the original method, KGF-9 was synthesized by dissolving Pb(NO_3_)_2_ and H_2_tadt as precursors in a water/acetone mixed solvent and heating the mixture in an autoclave for 48 h. The structure contains two-dimensional sheets composed of Pb and S, which are bridged by five-membered rings. N_2_ gas adsorption–desorption isotherms revealed a nonporous structure typical of bulk materials and a specific surface area of less than 1 m^2^ g^–1^, as determined by N_2_ gas adsorption. KGF-9 can absorb visible light with wavelengths as long as ∼ 500 nm without requiring surface modification, exhibiting an estimated bandgap of 2.5 eV (Figure 3b). The conduction-band minimum of KGF-9 is positioned at a more negative potential than those required to reduce CO_2_ to formic acid, CO, methanol, or methane, suggesting that it could theoretically function as a photocatalyst for CO_2_ reduction.

Using the obtained KGF-9, the photocatalytic activity for CO_2_ reduction was evaluated in a dimethyl sulfoxide (DMSO) solution containing 1,3-dimethyl-2-phenyl-2,3-dihydro-1*H*-benzimidazole (BIH). Here, BIH acts as a sacrificial agent, serving dual roles as an electron donor and a proton donor. As a result, KGF-9 could convert CO_2_ to formate without the assistance of metal nanoparticles or molecular metal complex catalysts. Notably, no H_2_, often observed as a byproduct in CO_2_ reduction reactions, was detected, and the selectivity to formate production exceeded 99%. In addition, the use of microwaves during the synthesis of KGF-9 led to high-quality samples having fibrous morphology with fewer defects, different from the original rod-shaped KGF-9.^33^ The change in the apparent quantum yield for the CO_2_-to-formate conversion over KGF-9 followed the change in the UV–vis diffuse-reflectance spectrum of KGF-9 (Figure 3b), indicating that the reaction occurred via light absorption of KGF-9. A maximum apparent quantum yield of 25% (at 400 nm) was obtained under the optimal conditions. This value is approximately 10 times higher than those obtained by conventional methods and represents the highest value reported for a heterogeneous photocatalyst capable of selectively converting CO_2_ to formate. High-rate, selective CO_2_ electrochemical reduction was also achieved even in aqueous solution using the fibrous KGF-9 fixed onto a conductive carbon support. During the electrolysis, the KGF-9 cathode underwent a structural change to a PbCO_3_/Pb_3_(CO_3_)_2_(OH)_2_ mixed phase, which functioned as active species for CO_2_-to-formate conversion. Interestingly, the performance of PbCO_3_ and Pb_3_(CO_3_)_2_(OH)_2_ individually was inferior to that of KGF-9.

Theoretical calculations showed that the superior properties of KGF-9 originate from the existence of surface tadt ligands and the resultant uneven surface, in which −SH groups attached to the ligand play a critical role during the selective CO_2_ conversion.^34^ A similar calculation was conducted for PbS, a typical inorganic crystal. Figure 3c shows that Pb in KGF-9 has a distorted S/N coordination and interacts with the π-conjugated ligand, whereas Pb in PbS adopts an octahedral Pb–S coordination. In KGF-9, the highest occupied states are localized near the surface and can contribute to CO_2_ reduction, while in PbS, they are confined within the bulk and thus inactive in catalysis. That is, the use of molecular anions to create unique catalytic active sites on the crystal surface of KGF-9 was found to contribute to the realization of efficient CO_2_ reduction. Theoretical calculation results for KGF-9 also imply that CPs/MOFs formed by anionic ligands, which could provide hydrogen (e.g., −SH or −NH_2_), may achieve the same functionality as KGF-9. Indeed, a Sn^2+^-based MOF of [Sn^II^_2_(Httc)_2_·MeOH]*_n_* (KGF-10; H_3_ttc = 1,3,5-triazine-2,4,6-trithiol trithiocyanuric acid, MeOH = methanol) could convert CO_2_ into formate with an apparent quantum yield of 9.8% at 400 nm and >99% selectivity.^35^ A Pb^2+^-based CP that consists of 3-amino-5-mercapto-1,2,4-triazole and acetate as anionic species has also been found to act as a selective CO_2_ reduction photocatalyst, although it is only UV-driven.^36^

As such, inorganic compounds that contain reactive molecular ions may provide active sites suitable for small-molecule conversion reactions. Examples include those in which proton-coupled electron-transfer play an essential role, such as the water oxidation and CO_2_ reduction reactions. The presence of active sites in such inorganic compounds was also suggested from a recent finding of a new CO_2_ reduction catalyst that consisted of ″iron rust.″^37^

## 4 Summary and Outlook

This Perspective provides an overview of anion-controlled inorganic catalysts for ammonia synthesis, water oxidation, and CO_2_ reduction. Regarding ammonia synthesis, oxyhydrides, which directly react with H_2_ gas as a reactant to promote ammonia synthesis, have been shown to be superior catalyst supports compared with conventional oxides. The involvement of labile H^–^ ions in the reaction process offers unique reaction pathways (e.g., the hydrogen-based Mars–van Krevelen mechanism) that are distinct from those of oxide supports and contribute to good ammonia synthesis activity. The ultimate goal in the field of ammonia synthesis catalysts is to establish a sustainable ammonia synthesis technology with low energy consumption and minimal environmental impact. To achieve this, from the perspective of catalyst material development, it is crucial not only to develop high-performance catalysts that operate under low-temperature and low-pressure (i.e., ambient pressure) conditions but also to reduce catalyst production costs by avoiding the use of rare metals. As introduced in this Perspective, catalysts utilizing anions have the potential to contribute to these objectives, and further advancements in research are anticipated in the future.

By contrast, F, which is chemically stable and has the highest electronegativity among the elements, is useful as a constituent anion for OER electrocatalysts because the strong electron-withdrawing nature of F can render the active metal in a more desirable electron-deficient state. The rutile-type oxyfluoride Co_0.5_Fe_0.5_O_0.5_F_1.5_ was shown to outperform most of the reported Co-based OER catalysts in terms of overpotential, Tafel slope, mass activity, and stability. To date, metal oxides have been considered representative OER catalysts. However, as discussed in this Perspective, some oxyfluorides and oxychlorides can exhibit OER catalytic activity equal to or even exceeding that of oxides, although their current performance is not yet satisfactory for practical applications. Further improvements are therefore required in terms of reaction rate, durability, and production cost to replace existing OER electrocatalysts that use precious metals. Additionally, beyond their application as OER electrocatalysts, it will also be important in the future to develop nanoparticulate high-performance OER catalysts that work under nonelectrochemical conditions and contribute to artificial photosynthesis, which is of importance as future technology for clean H_2_ production. Notably, OER electrocatalysts are, in general, useful as ″cocatalysts″ in overall water splitting by particulate semiconductor photocatalysts.^38^ In understanding catalysts that operate in such nonelectrochemical environments, in addition to leveraging well-established computational approaches, a recently developed technique that enables to measure the catalyst potential under operating conditions^39^ may also prove effective.

Another example of mixed-anion compounds that participate in chemical reactions is the layered oxyfluoride La_1.2_Sr_1.8_Mn_2_O_7−δ_F_2_, which has been used as a cathode material for F^–^-ion batteries.^40^ In this material, O–O bonds are formed reversibly in the crystal structure as a result of the intercalation of excess F^–^ ions during the charging process, which results in a high reversible capacity.

A common challenge in mixed-anion catalyst systems is the extreme difficulty of their synthesis. Consequently, freely controlling key factors that affect catalytic activity, such as specific surface area and particle morphology, is not as straightforward as is in the case of metal oxides.^4^ For oxyhydrides and oxyfluorides, mechanochemical synthesis^18,41^ and microwave-assisted synthesis^42^ have been reported in recent years, suggesting that improvements in the synthesis methods may lead to the development of higher-performance catalysts in the future. In addition to the improvement of synthesis method, understanding the structure–activity relationship in mixed-anion catalysts is of importance. While it is known that defects, vacancies, and lattice distortion in metal oxides affect their catalytic activity, however, the impact of the structural properties on catalytic activity of mixed-anion compounds has not been elucidated enough. The importance of anion vacancy in activating N_2_ during ammonia synthesis has been highlighted for Ba–Si orthosilicate oxynitride-hydride very recently,^43^ although quantitative understanding of the structure–activity relationship requires further investigation. To understand the nature of defects/vacancies in mixed-anion compounds, theoretical calculations should be at play,^44,45^ along with operando spectroscopy measurements that could visualize the change in the oxidation state of catalytically active sites and detect reaction intermediates on the catalyst surface.^46,47^

Certain CPs with metal–sulfur bonds (i.e., KGF-9 and KGF-10) can exhibit good photocatalytic activity for CO_2_-to-formate conversion because of their unique surface structure formed by molecular anion species, which is unattainable with ordinary inorganic materials composed of monatomic ions. Catalysts that selectively promote CO_2_ reduction are well-known, with homogeneous catalysts such as metal complexes being representative examples. Both Pb and Sn are metals used as solid catalysts for CO_2_ reduction, but they are not necessarily suitable for “selective reduction” targeting specific desired products. Although numerous metal complexes have been reported to function as active catalysts or photocatalysts for CO_2_ reduction, the literature includes no known examples incorporating Pb or Sn as the central elements. The CP/MOF photocatalysts KGF-9 and KGF-10, which exhibit high activity in visible-light-driven CO_2_ reduction reactions, can be positioned as intermediates between molecules and inorganic solids (such as oxides composed of single-atom units). That is, these catalysts represent examples where solidifying molecular structures has enabled functionalities that cannot be achieved by the molecules alone. Currently, these CP/MOF photocatalysts require reductants such as BIH to drive CO_2_ reduction reactions. However, with further elucidation of the CO_2_ reduction mechanisms including the structure of active sites and reaction intermediates as well as the development of more advanced catalyst design principles, the realization of light energy conversion-type CO_2_ reduction reactions is anticipated.
